# Early wound morbidity and clinical outcomes associated with P4HB mesh compared to permanent synthetic mesh in umbilical and small to medium, routine ventral hernia repairs

**DOI:** 10.3389/fsurg.2023.1280991

**Published:** 2023-10-10

**Authors:** Corey R. Deeken, Michael J. Rosen, Benjamin K. Poulose, Kasia Bradbury, Li-Ching Huang, Jianing Ma, Amit Badhwar

**Affiliations:** ^1^ Covalent Bio, LLC, St. Louis, MO, United States; ^2^ Cleveland Clinic Center for Abdominal Core Health, Cleveland Clinic, the Cleveland Clinic Foundation, Digestive Diseases and Surgery Institute, Cleveland, OH, United States; ^3^ Center for Abdominal Core Health, The Ohio State Wexner Medical Center, Columbus, OH, United States; ^4^ BD Interventional (Surgery), Warwick, RI, United States; ^5^ Vanderbilt University Medical Center, Nashville, TN, United States; ^6^ Center for Biostatistics, Department of Biomedical Informatics, The Ohio State University Wexner Medical Center, Columbus, OH, United States

**Keywords:** hernia recurrence, poly-4-hydroxybutyrate, P4HB, surgical site infection (SSI), umbilical hernia, ventral hernia

## Abstract

**Background:**

Permanent synthetic meshes such as polypropylene (PP) have been utilized for hernia repair for decades, but concerns remain regarding potential long-term, mesh-related complications. A resorbable polymer such as poly-4-hydroxybutyrate (P4HB) represents an alternative with high initial strength, that gradually resorbs, leaving an abdominal wall that is at least as strong as it would be in its native state. We aimed to compare early wound morbidity and clinical outcomes associated with P4HB to traditional, permanent PP in umbilical and small to medium, routine ventral hernias using data from the Abdominal Core Health Quality Collaborative (ACHQC).

**Methods:**

Inclusion criteria for the umbilical cohort included: all Centers for Disease Control and Prevention (CDC) wound classes, all Ventral Hernia Working Group (VHWG) hernia grades, and hernia defects <3 cm. The small to medium, routine ventral hernia cohort was limited to CDC class I wounds, VHWG hernia grades I and II, and hernia defects <5 cm. The study group was comprised of P4HB meshes; the comparator group was an aggregate of PP meshes. Clinical outcomes were assessed at 30 days.

**Results:**

There was no significant difference in early wound morbidity, readmission, or reoperation between the P4HB and PP cohorts. A small number of patients experienced SSO, with ≤4% requiring procedural intervention. None of the patients (0% in all cases) experienced skin/soft tissue necrosis, infected seroma, infected hematoma, exposed/contaminated/infected mesh, enterocutaneous fistula, graft failure, or pain requiring intervention at 30-days. However, P4HB was associated with significantly greater operative time, length of stay, and use of myofascial release compared to PP (*p* < 0.05 in all cases).

**Conclusions:**

Short-term clinical outcomes associated with resorbable P4HB mesh are comparable to permanent synthetic PP mesh in umbilical and small to medium, routine ventral hernia repairs, despite significant differences in operative time and length of stay. Longer-term follow-up is needed to expand on the clinical relevance of these short-term findings.

## Introduction

Poly-4-hydroxybutyrate (P4HB) is a biologically produced, fully resorbable synthetic polymer that has been used to construct meshes for hernia repair applications (1–3). Bench and preclinical studies have established the long-term strength and durability of P4HB, and clinical studies have extended these findings, documenting outcomes comparable to other mesh materials (4).

P4HB mesh has been utilized in a wide variety of patient populations, but a recent scoping review identified a knowledge gap in umbilical and small to medium-sized, routine ventral hernias (4). Permanent synthetic meshes are commonly utilized in these applications (5–9). However, the possibility of long-term, mesh-related complications such as seroma, chronic pain, infection, fistula, and bowel obstruction must be considered due to the permanent nature of these materials (10).

Meshes constructed of a fully resorbable polymer such as P4HB represent a potential advantage over permanent polymers due to their temporary status within the abdominal wall, both in terms of reducing possible mesh-related complications, as well as in decreasing the complexity of subsequent abdominal procedures (10, 11). However, it has been suggested that the resorption of these materials may provoke a more aggressive, early host tissue response in the form of increased macrophage activation (12) and inflammation (13), potentially leading to higher rates of early wound morbidity compared to permanent synthetic meshes (14). To date, this concept has only been assessed in a cohort of complex cases involving clean-contaminated and contaminated wounds, which contributed a number of confounding factors to the analysis and prevented definitive conclusions (14).

Thus, two important knowledge gaps currently exist in the scientific literature surrounding P4HB mesh use in hernia repair applications, namely a lack of clinical data in umbilical and small to medium-size ventral hernia populations, as well as an evaluation of the early wound morbidity associated with less complex or “routine” cases. The current study was designed to focus on these populations, eliminating a number of confounding factors, in an effort to better understand potential differences between resorbable, monofilament P4HB mesh compared to permanent, monofilament polypropylene mesh.

## Methods

A retrospective analysis was completed utilizing data collected prospectively through the Abdominal Core Health Quality Collaborative (ACHQC) database (formerly the Americas Hernia Society Quality Collaborative—AHSQC) (15). The ACHQC is a voluntary, national database that is prospectively maintained for the purpose of improving abdominal core health through a continuous quality improvement process. At the time of this study, data were available within the ACHQC for *n* = 102,084 total patients (*n* = 43,191 ventral hernia patients) contributed by *n* = 424 surgeons from academic, community, and academic-affiliated hospital settings.

The ACHQC database was queried for patients undergoing umbilical or small to medium, routine ventral hernias between January 2012 and September 2022. The inclusion criteria for the umbilical hernia cohort included: all Centers for Disease Control and Prevention (CDC) wound classes (16) and all Ventral Hernia Working Group (VHWG) hernia grades (17), as well as hernia defects <3 cm. Although this cohort is entitled “umbilical hernia”, ventral hernias <3 cm were also included as a surrogate for less complex umbilical hernias. The inclusion criteria for the small to medium, routine ventral hernia cohort were slightly different and limited to CDC wound class I wounds, VHWG hernia grades I and II, and hernia defects <5 cm. In this cohort, the term “small to medium, routine ventral hernia” was not defined according to a validated scale. Rather, the term was intended to differentiate this cohort from more complex cases described in the literature (e.g., CDC wound classes II, III, and IV; VHWG hernia grades III and IV; hernias >5 cm, etc.). For both cohorts, the study group was comprised of poly-4-hydroxybutyrate meshes (Phasix™ Mesh or Phasix™ ST Mesh—collectively “P4HB”; Becton, Dickinson, and Company, Warwick, RI), while the comparator group was comprised of an aggregate of permanent synthetic polypropylene meshes (Bard™ Mesh, Bard™ Soft Mesh, Ventralex™ Hernia Patch, Ventralex™ ST Hernia Patch, Ventralight™ ST Mesh, Ventrio™ Hernia Patch, and Ventrio™ ST Hernia Patch—collectively “PP”; Becton, Dickinson, and Company, Warwick, RI).

Clinical outcomes were assessed at 30 days. Primary outcomes were defined as perioperative complications, including immediate hernia recurrence, surgical site infection (SSI), surgical site occurrence (SSO) and surgical site occurrence with procedural intervention (SSOPI). Hernia recurrence was assessed by physical exam and/or computed tomography (CT) scan when possible. A pragmatic definition of recurrence was adapted from Krpata et al. (18) SSI were defined according to the CDC, (16) and SSO included SSI, wound cellulitis, non-healing incisional wound, fascial disruption, skin/soft tissue ischemia or necrosis, serous or purulent wound drainage, stitch abscess, seroma, hematoma, infected or exposed mesh, or enterocutaneous fistula. Procedural interventions for SSOPI included wound opening, wound debridement, suture excision, percutaneous drainage, and partial/complete mesh removal. Secondary outcomes included operative time, length of stay, hospital readmission, and reoperation.

A propensity score model (PSM) and matching algorithms were implemented to address potential treatment choice bias. The PSM is based on the probability of assignment to the treatment group, and was selected due to a good balance on the covariates, rather than Mahalanobis distance matching, which is based on covariate values (19). Propensity score matches were generated by matching patients receiving P4HB mesh with those receiving PP mesh. An *a priori* approach was used to select the clinical variables to include in the PSM based on known risk factors that can impact the outcome measure. The variables included in the PSM were: VHWG grade, hernia width, anti-platelet medications, age capped at 90, operation approach, number of prior hernia repairs, race, hypertension, concomitant procedure, ASA class, BMI capped 15–60, diabetes mellitus, and current smoker. One matched control was selected for each case. Logistic regression was used to estimate the propensity scores. Inverse probability weighting (IPWT) was not performed, as the current study design did not include a weighted regression approach. Standardized mean differences (SMD) were used to measure the balance following propensity score matching. An SMD of 0.1 was considered the threshold for good balance, and 0.2 was considered acceptable. Nearest neighbor matching without replacement was utilized as it is the most common form of greedy matching with similar performance as optimal matching (20); no caliper was used. Pearson's Chi-squared test was used to compare categorical variables between P4HB and PP, while Wilcoxon rank sum test was used to compare continuous variables. A *p*-value < 0.05 was considered statistically significant.

## Results

### Umbilical hernia cohort

In the umbilical hernia cohort, *n* = 122 patients were evaluated in each group (Table 1). After propensity score matching, the P4HB and PP groups were comprised of several mesh products (Monofilament P4HB Meshes: *n* = 76 Phasix™ Mesh and *n* = 46 Phasix™ ST Mesh and Monofilament PP Meshes: *n* = 4 Bard™ Mesh, *n* = 25 Bard™ Soft Mesh, *n* = 4 Ventralex™ Hernia Patch, *n* = 42 Ventralex™ ST Hernia Patch, *n* = 43 Ventralight™ ST Mesh, *n* = 0 Ventrio™ Hernia Patch, and *n* = 4 Ventrio™ ST Hernia Patch, respectively.) Patients were primarily White and non-Hispanic (P4HB: 73.8% vs. PP: 73.8%; *p* > 0.05) with similar age (P4HB: 49.0 (38.2–57.8) years vs. PP: 47.0 (38.0–59.0) years; median (IQR); *p* > 0.05), BMI (P4HB: 29.4 (24.7–34.8) kg/m^2^ vs. PP: 29.7 (26.8–34.6) kg/m^2^; median (IQR); *p* > 0.05), comorbidities, wound classification, and number of prior repairs (P4HB: 0.0 (0.0–0.0) prior repairs vs. PP: 0.0 (0.0–0.0) prior repairs; median (IQR); *p* > 0.05); divided almost equally among males (P4HB: 56.6% vs. PP: 50.8%; *p* > 0.05) and females (P4HB: 43.4% vs. PP: 49.2%; *p* > 0.05).

**Table 1:** Patient demographics.

	Umbilical hernia		Small to medium, routine ventral hernia	
	Phasix	PP	Phasix	PP
Number of patients	122	122	235	235
Sex, *n* (%)				
Male	69 (56.6%)	62 (50.8%)	133 (56.6%)	129 (54.9%)
Female	53 (43.4%)	60 (49.2%)	102 (43.4%)	106 (45.1%)
Ethnicity				
American Indian or Alaskan Native	3 (2.5%)	1 (0.8%)	3 (1.3%)	1 (0.4%)
Asian or Pacific Islander	1 (0.8%)	0 (0.0%)	2 (0.9%)	2 (0.9%)
Black, not of Hispanic origin	10 (8.2%)	18 (14.8%)	20 (8.5%)	33 (14.0%)
Hispanic	16 (13.1%)	11 (9.0%)	24 (10.2%)	19 (8.1%)
White, not of Hispanic origin	90 (73.8%)	90 (73.8%)	184 (78.3%)	176 (74.9%)
Middle Eastern	1 (0.8%)	2 (1.6%)	2 (0.9%)	4 (1.7%)
Not indicated	1 (0.8%)	0 (0.0%)	0 (0.0%)	0 (0.0%)
Age, years ± SD	49.0 ± 13.7	48.7 ± 14.5	52.9 ± 14.7	53.4 ± 14.3
Median	49.0	47.0	54.0	55.0
IQR	38.2–57.8	38.0–59.0	41.0–65.0	43.0–64.0
BMI, kg/m^2^, mean ± SD	31.0 ± 8.2	31.2 ± 7.0	30.3 ± 6.3	30.1 ± 6.3
Median	29.4	29.7	29.2	29.3
IQR	24.7–34.8	26.8–34.6	26.3–33.5	26.4–32.8
Comorbidities				
Smoking (current within 1 month)	13 (10.7%)	13 (10.7%)	20 (8.5%)	19 (8.1%)
Diabetes mellitus	12 (9.8%)	13 (10.7%)	18 (7.7%)	16 (6.8%)
Hypertension	39 (32.0%)	40 (32.8%)	71 (30.2%)	69 (29.4%)
Wound classification				
Clean	104 (85.2%)	108 (88.5%)	235 (100.0%)	235 (100.0%)
Clean—contaminated	13 (10.7%)	14 (11.5%)	0 (0.0%)	0 (0.0%)
Contaminated	4 (3.3%)	0 (0.0%)	0 (0.0%)	0 (0.0%)
Dirty	1 (0.8%)	0 (0.0%)	0 (0.0%)	0 (0.0%)
VHWG				
1	54 (44.3%)	55 (45.1%)	124 (52.8%)	124 (52.8%)
2	46 (37.7%)	45 (36.9%)	111 (47.2%)	111 (47.2%)
3	21 (17.2%)	21 (17.2%)	0 (0.0%)	0 (0.0%)
4	1 (0.8%)	1 (0.8%)	0 (0.0%)	0 (0.0%)
Number of prior hernia repairs, mean ± SD	0.2 ± 0.5	0.2 ± 0.6	0.3 ± 0.6	0.3 ± 0.6
Median	0.0	0.0	0.0	0.0
IQR	0.0–0.0	0.0–0.0	0.0–0.0	0.0–0.0

As shown in Table 2, the majority of patients in the umbilical hernia cohort underwent open (P4HB: 50.0% vs. PP: 45.1%; *p* > 0.05) or robotic repairs (P4HB: 42.6% vs. PP: 36.1%; *p* > 0.05) with sublay mesh placement (P4HB: 72.1% vs. PP: 92.6%; *p* > 0.05). Within the sublay category, significantly more retrorectus or retromuscular repairs were performed in the P4HB cohort (43.2%) compared to the PP cohort (10.6%, *p* < 0.05). Conversely, significantly more preperitoneal and intraperitoneal sublay repairs were performed in the PP cohort compared to the P4HB cohort (preperitoneal: P4HB: 17.0% and PP: 31.0%; intraperitoneal: P4HB: 39.8% and PP: 58.4%; *p* < 0.05 in all cases). Fixation was utilized in greater than 90% of both P4HB and PP mesh groups.

**Table 2:** Operative details.

	Umbilical hernia		Small to medium, routine ventral hernia	
	Phasix	PP	Phasix	PP
Number of patients	122	122	235	235
Operative time	*		*	
0–59	42 (34.4%)	56 (45.9%)	59 (25.1%)	74 (31.5%)
60–119	36 (29.5%)	46 (37.7%)	64 (27.2%)	84 (35.7%)
120–179	35 (28.7%)	11 (9.0%)	75 (31.9%)	53 (22.6%)
180–239	8 (6.6%)	3 (2.5%)	23 (9.8%)	17 (7.2%)
240+	1 (0.8%)	6 (4.9%)	14 (6.0%)	7 (3.0%)
Length of stay, days ± SD	1.7 ± 3.8*	0.4 ± 1.2	2.0 ± 4.9*	1.0 ± 1.7
Median	0.0	0.0	1.0	0.0
IQR	0.0–1.0	0.0–0.0	0.0–2.0	0.0–1.0
Operative technique				
Laparoscopic	8 (6.6%)	18 (14.8%)	23 (9.8%)	36 (15.3%)
Laparoscopy-assisted ventral hernia repair	0 (0.0%)	2 (1.6%)	0 (0.0%)	4 (1.7%)
MIS convert to open	0 (0.0%)	2 (1.6%)	2 (0.9%)	1 (0.4%)
Open	61 (50.0%)	55 (45.1%)	131 (55.7%)	119 (50.6%)
Robotic	52 (42.6%)	44 (36.1%)	77 (32.8%)	71 (30.2%)
Robotic-assisted ventral hernia repair	1 (0.8%)	1 (0.8%)	2 (0.9%)	4 (1.7%)
Procedure category				
Incisional	26 (21.3%)	27 (22.1%)	120 (51.1%)	134 (57.0%)
Parastomal	2 (1.6%)	2 (1.6%)	4 (1.7%)	3 (1.3%)
Epigastric (primary ventral)	20 (16.4%)*	8 (6.6%)	49 (20.9%)*	24 (10.2%)
Umbilical (primary ventral)	80 (65.6%)	88 (72.1%)	81 (34.5%)	82 (34.9%)
Spigelian	3 (2.5%)	2 (1.6%)	4 (1.7%)	3 (1.3%)
Lumbar	0 (0.0%)	0 (0.0%)	0 (0.0%)	2 (0.9%)
Diastasis recti	7 (5.7%)	2 (1.6%)	22 (9.4%)*	3 (1.3%)
Mesh location	*		*	
Inlay	2 (1.6%)	2 (1.6%)	4 (1.7%)	6 (2.6%)
Onlay	32 (26.2%)	7 (5.7%)	49 (20.9%)	16 (6.8%)
Sublay	88 (72.1%)	113 (92.6%)	182 (77.4%)	213 (90.6%)
Sublay retrorectus or retromuscular	38 (43.2%)*	12 (10.6%)	105 (57.7%)*	63 (29.6%)
Sublay preperitoneal	15 (17.0%)*	35 (31.0%)	19 (10.4%)*	70 (32.9%)
Sublay intraperitoneal	35 (39.8%)*	66 (58.4%)	62 (34.1%)*	99 (46.5%)
Myofascial release	37 (30.3%)*	14 (11.5%)	106 (45.1%)*	75 (31.9%)
External oblique	0 (0.0%)	1 (7.1%)	4 (3.8%)	3 (4.0%)
Transversus abdominis	4 (10.8%)	1 (7.1%)	23 (21.9%)	24 (32.0%)
Anterior rectus sheath	1 (2.7%)	0 (0.0%)	1 (1.0%)	3 (4.0%)
Posterior rectus sheath	36 (97.3%)	14 (100.0%)	101 (96.2%)*	64 (85.3%)
Fixation used	113 (92.6%)	111 (91.0%)	218 (92.8%)	206 (87.7%)
ASA class				
1	20 (16.4%)	17 (13.9%)	35 (14.9%)	27 (11.5%)
2	64 (52.5%)	68 (55.7%)	126 (53.6%)	143 (60.9%)
3	36 (29.5%)	35 (28.7%)	72 (30.6%)	63 (26.8%)
4	2 (1.6%)	2 (1.6%)	2 (0.9%)	2 (0.9%)
5	0 (0.0%)	0 (0.0%)	0 (0.0%)	0 (0.0%)
Hernia width, cm ± SD	2.8 ± 1.5*	2.7 ± 2.3	3.7 ± 2.2	3.8 ± 2.5
Median	2.0	2.0	3.0	3.0
IQR	2.0–3.0	2.0–3.0	3.0–4.0	2.0–4.0
Concomitant procedures^a^	16 (13.1%)	18 (14.8%)	21 (8.9%)*	39 (16.6%)

Meshes were placed in multiple sublay locations in several patients and were counted in all of the applicable categories, resulting in a sum of percentages greater than 100%.

* *p* < 0.05.

^a^ Concomitant procedures included: colorectal, foregut, endocrine, hepatobiliary, pancreatic, small intestine, hernia, obstetric/gynecologic, soft tissue/plastics, and urologic procedures.

Patients in the P4HB group reported significantly longer operative time (P4HB: 36.1% ≥ 2 h vs. PP: 16.4% ≥ 2 h; *p* < 0.001), significantly longer length of stay (P4HB: 1.7 ± 3.8 days (median: 0.0; IQR: 0.0–1.0) vs. PP: 0.4 ± 1.2 days (median: 0.0; IQR: 0.0–0.0); *p* < 0.001), more epigastric hernias (P4HB: 16.4% vs. PP: 6.6%; *p* = 0.016), and more procedures requiring myofascial release (P4HB: 30.3% vs. PP: 11.5%; *p* < 0.001) compared to those receiving PP mesh (Table 2). There were no significant differences between P4HB and PP with regard to any of the short-term (30-day) postoperative complications evaluated in this study (Table 3), particularly hospital readmission, hernia recurrence, SSI, SSO, and SSOPI (*p* > 0.05 in all cases). A small number of patients experienced non-healing incisional wounds, fascial disruption, wound/serous drainage, seroma, and hematoma, among others. None of the patients in the umbilical hernia cohort experienced hernia recurrence, SSI, skin/soft tissue necrosis/ischemia, wound cellulitis, infected seroma, infected hematoma, exposed/contaminated/infected mesh, enterocutaneous fistula, graft failure, or pain requiring intervention at 30-days. As shown in Table 3, a small number of patients in each cohort required readmission (P4HB: 3.3%; PP: 2.8%, *p* > 0.05). In the P4HB cohort, readmission occurred due to wound, gastrointestinal, and unknown complications (*n* = 1 in each category) and in the PP cohort due to pain (*n* = 1) and gastrointestinal complications (*n* = 2). However, none of the patients in either cohort required reoperation during the follow-up period (*n* = 0 in all of the following categories: unrecognized bowel injury, major wound complication, postoperative bleeding, early recurrence, bowel obstruction, unrelated intraabdominal pathology, mesh excision, hernia at new ostomy site, or port site hernia).

**Table 3:** 30-day clinical outcomes.

		Umbilical hernia		Small to medium, routine ventral hernia	
		Phasix	PP	Phasix	PP
Number of patients		122	122	235	235
Number of evaluated patients		92 (75.4%)*	106 (86.9%)	174 (74.0%)*	193 (82.1%)
Follow-up time, days ± SD		27.7 ± 10.2*	21.5 ± 10.3	26.7 ± 10.7*	22.2 ± 10.2
** **Median		29.0	18.5	29.0	20.0
** **IQR		20.0–35.0	15.0–28.0	17.0–35.0	15.0–29.0
Hernia recurrence		0 (0.0%)	0 (0.0%)	0 (0.0%)	0 (0.0%)
Surgical site infection		0 (0.0%)	0 (0.0%)	2 (1.1%)	1 (0.5%)
Surgical site occurrences		6 (6.5%)	5 (4.7%)	11 (6.3%)	19 (9.8%)
SSO requiring procedural intervention		3 (3.3%)	1 (0.9%)	7 (4.0%)	4 (2.1%)
Readmission		3 (3.3%)	3 (2.8%)	7 (4.1%)	6 (3.1%)
Readmission reason					
Pain		0	1	1	3
Prosthetic related complication		0	0	0	1
Wound complication		1	0	2	1
Bleeding complication		0	0	0	1
Thrombotic complication (non-cardiac)		0	0	0	0
Gastrointestinal complication		1	2	3	0
Cardiac complication		0	0	0	0
Respiratory complication		0	0	1	0
Reoperation		0 (0.0%)	0 (0.0%)	2 (1.1%)	2 (1.0%)
Reoperation reason					
Unrecognized bowel injury		0	0	0	0
Major wound complication		0	0	0	1
Postoperative bleeding		0	0	2	1
Early recurrence		0	0	0	0
Bowel obstruction		0	0	0	0
Unrelated intra-abdominal pathology		0	0	0	0
Mesh excision		0	0	0	0
Hernia at new ostomy site		0	0	0	0
Port site hernia		0	0	0	0
Complication types of SSO	Exposed synthetic mesh	0	0	0	0
	Contaminated biologic mesh	0	0	0	0
	Contaminated synthetic mesh	0	0	0	0
	Infected biologic mesh	0	0	0	0
	Infected synthetic mesh	0	0	0	0
	Wound cellulitis	0	0	0	3
	Non-healing incisional wound	0	1	0	0
	Fascial disruption	1	0	0	0
	Skin or soft tissue ischemia	0	0	0	2
	Wound serous drainage	1	3	2	3
	Seroma	3	0	8	10
	Infected seroma	0	0	0	0
	Hematoma	2	1	2	1
	Unspecified surgical site occurrence	0	0	0	2
	Pulmonary embolism	0	0	1	0
	Sepsis	0	0	0	1
	Urinary tract infection	1	1	1	2
	Post-op bleeding transfusion	0	0	3	1
	Other	1	2	3	0

Percentages for the individual complications were not included in order to simplify the interpretation of the data and avoid the possibility of misinterpretation as the sum of the SSOPI exceeds 100% in several categories.

* *p* < 0.05.

### Small to medium, routine ventral hernia cohort

In the small to medium, routine ventral hernia cohort, *n* = 235 patients were evaluated in each group (Table 1). After propensity score matching, the P4HB and PP groups were comprised of several mesh types (P4HB: *n* = 148 Phasix™ Mesh and *n* = 87 Phasix™ ST Mesh and PP: *n* = 19 Bard™ Mesh, *n* = 78 Bard™ Soft Mesh, *n* = 15 Ventralex™ Hernia Patch, *n* = 48 Ventralex™ ST Hernia Patch, *n* = 69 Ventralight™ ST Mesh, *n* = 2 Ventrio™ Hernia Patch, and *n* = 4 Ventrio™ ST Hernia Patch, respectively.) As with the umbilical hernia cohort, patients in the small to medium, routine ventral hernia cohort were again primarily White and non-Hispanic (P4HB: 78.3% vs. PP: 74.9%; *p* > 0.05) with similar age (P4HB: 54.0 (41.0–65.0) years vs. PP: 55.0 (43.0–64.0) years; median (IQR); *p* > 0.05), BMI (P4HB: 29.2 (26.3–33.5) kg/m^2^ vs. PP: 29.3 (26.4–32.8) kg/m^2^; median (IQR); *p* > 0.05), comorbidities, wound classification, and number of prior repairs (P4HB: 0.0 (0.0–0.0) prior repairs vs. PP: 0.0 (0.0–0.0) prior repairs; median (IQR); *p* < 0.05); divided almost equally among males (P4HB: 56.6% vs. PP: 54.9%; *p* > 0.05) and females (P4HB: 43.4% vs. PP: 45.1%; *p* > 0.05).

As shown in Table 2, the majority of patients in the small to medium, routine ventral hernia cohort underwent open (P4HB: 55.7% vs. PP: 50.6%; *p* > 0.05) or robotic repairs (P4HB: 32.8% vs. PP: 30.2%; *p* > 0.05) with sublay mesh placement (P4HB: 77.4% vs. PP: 90.6%; *p* > 0.05). Within the sublay category, significantly more retrorectus or retromuscular repairs were performed in the P4HB cohort (57.7%) compared to the PP cohort (29.6%, *p* < 0.05). Conversely, significantly more preperitoneal and intraperitoneal sublay repairs were performed in the PP cohort compared to the P4HB cohort (preperitoneal: P4HB: 10.4% and PP: 32.9%; intraperitoneal: P4HB: 34.1% and PP: 46.5%; *p* < 0.05 in all cases). Meshes were placed in multiple sublay locations in several patients and were counted in all of the applicable categories, resulting in a sum of percentages greater than 100% (Table 2). Fixation was utilized in greater than 85% of both P4HB and PP mesh groups.

Patients in the P4HB group reported significantly longer operative time (P4HB: 47.7% ≥ 2 h vs. PP: 32.8% ≥ 2 h; *p* = 0.022), significantly longer length of stay (P4HB: 2.0 ± 4.9 days (median: 1.0; IQR: 0.0–2.0) vs. PP: 1.0 ± 1.7 days (median: 0.0; IQR: 0.0–1.0); *p* = 0.020), more epigastric hernias (P4HB: 20.9% vs. PP: 10.2%; *p* = 0.001), significantly more procedures requiring myofascial release (P4HB: 45.1% vs. PP: 31.9%; *p* = 0.003) and significantly fewer concomitant procedures (P4HB: 8.9% vs. PP: 16.6%; *p* = 0.013) compared to those receiving PP mesh (Table 2). There were no significant differences between P4HB and PP with regard to any of the short-term (30-day) postoperative complications evaluated in this study (Table 3), particularly hospital readmission, hernia recurrence, SSI, SSO, and SSOPI (*p* > 0.05 in all cases). A small number of patients experienced wound cellulitis, skin/soft tissue ischemia, wound/serous drainage, seroma, and hematoma, among others. None of the patients in the small to medium, routine ventral hernia cohort experienced hernia recurrence, non-healing incisional wound, fascial disruption, skin/soft tissue necrosis, infected seroma, infected hematoma, exposed/contaminated/infected mesh, enterocutaneous fistula, graft failure, or pain requiring intervention at 30-days. As shown in Table 3, a small number of patients in each cohort required readmission (P4HB: 4.1%; PP: 3.1%; *p* > 0.05). In the P4HB cohort, readmission occurred due to pain (*n* = 1), wound complications (*n* = 2), gastrointestinal complications (*n* = 3), and respiratory complications (*n* = 1). In the PP cohort, readmission occurred due to pain (*n* = 3) and prosthetic-related, wound, or bleeding complications (*n* = 1 in each category). Similarly, a small number of patients in each cohort required reoperation during the follow-up period (P4HB: 1.1%; PP: 1.0%; *p* > 0.05). In the P4HB cohort, reoperation occurred due to postoperative bleeding (*n* = 2) and in the PP cohort due to major wound complications and postoperative bleeding (*n* = 1 in each category).

## Discussion

Permanent synthetic meshes such as PP have been successfully utilized in hernia repair for several decades, but long-term, mesh-related complications remain concerning. Resorbable polymers such as P4HB gradually resorb, returning the abdominal wall to its native state without the presence of a foreign material over the long-term. However, there is some concern about the potential impact of these resorbable materials on the early host tissue response and subsequent wound morbidity related to increased macrophage activation (12) or inflammatory response (13). The current study was therefore designed to compare early wound morbidity and clinical outcomes associated with a fully resorbable hernia mesh material (P4HB) to a traditional, permanent material (PP) in umbilical and small to medium-sized, routine ventral hernias.

No significant differences were observed between the P4HB and PP cohort with regard to the primary outcomes of early wound morbidity or the secondary outcomes of readmission and reoperation (*p* > 0.05 in all cases). However, differences were observed between the P4HB and PP cohorts for the secondary outcomes of operative time and length of hospital stay. Resorbable P4HB meshes were associated with significantly longer operative times, significantly longer length of hospital stay, and significantly more frequent use of myofascial release compared to permanent PP mesh in both the umbilical hernia and small to medium, routine ventral hernia cohorts. This is likely attributed to aspects of the surgical techniques employed in these cases and demonstrates the “real-world” nature of the data obtained through the ACHQC. The majority of the P4HB meshes were implanted in the retrorectus/retromuscular tissue plane, while PP meshes were implanted predominantly in the intraperitoneal tissue plane. Additionally, ∼30%–40% of the cases in this study utilized a robotic technique (Table 2). It is well known that a robotic, retromuscular/preperitoneal repair (i.e., extended totally extraperitoneal—eTEP) is a much lengthier procedure than a robotic intraperitoneal mesh repair. P4HB repairs utilized myofascial release significantly more frequently than PP, even in the umbilical hernia cohort. The results of this study provide unique insight into how surgeons are utilizing P4HB mesh, namely via robotic, retromuscular repairs with myofascial release in small, uncomplicated hernias, leading to longer operative time and length of hospital stay. Despite “overtreating” these small hernias and the slightly higher number of more complex cases in the P4HB group in this study, early wound morbidity and clinical outcomes were comparable between P4HB and PP groups. The longer operative times and length of hospital stay reported in the P4HB cohort would otherwise favor the PP cohort, yet we did not observe any significant differences in clinical observations of wound occurrences, suggesting that the fully resorbable P4HB did not negatively impact early wound morbidity as speculated in previous studies involving complex patients with clean-contaminated or contaminated wounds (14).

The results of this study compare well with the published literature, including a pilot study involving P4HB mesh in small to medium-sized, routine ventral hernias in which Plymale et al. (21) reported 0.0% SSI, 19.4% SSO (primarily seroma) and 12.9% readmission at a median follow up of 414 days. In a retrospective case review of the National Surgery Quality Improvement (NSQIP) database, Wagner et al. (22) reported 11.8% SSI, 14.5% wound complication, and 11.8% readmission in CDC Class I (low-risk) ventral hernia repairs. However, both of these studies involved much larger defects and longer follow-up time than the current study, which likely contributed to the higher rates of wound morbidity observed in these studies relative to the current study.

Both cohorts of the current study exhibited similar hospital readmission rates at 30 days, regardless of mesh type or patient population. Patients in the P4HB cohort reported a 3.3% hospital readmission in the umbilical hernia cohort and 4.0% in the small to medium, routine ventral hernia cohort (*p* > 0.05). Similarly, patients in the PP cohort reported a 2.8% hospital readmission in the umbilical hernia cohort and 3.1% in the small to medium, routine ventral hernia cohort (*p* > 0.05). These findings are in agreement with the published literature which has reported 30-day readmission rates of ∼5%–13% in similar cohorts (23–27).

The current study is not without limitations, most notably its retrospective nature, focus on short-term, 30-day clinical outcomes, and the potential for selection bias inherent to data derived from a voluntary database. However, the data generated provide unique insight into the “real-world” use of these biomaterials in low-risk umbilical and ventral hernia repairs without the confounding factors that may have influenced wound morbidity in previously reported complex cases (14). Furthermore, the 30-day follow-up period represents a critical look into the potential contribution of resorbable materials to early wound morbidity. While the current data demonstrate comparable early wound morbidity between the P4HB and PP cohorts, future studies should be conducted with appropriate long-term follow-up to further develop the clinical relevance and implications of these early findings and support the long-term efficacy of P4HB mesh in routine hernia repair. Additionally, cost was not evaluated in this study, however, the economic impact of utilizing P4HB mesh is an important consideration and will be evaluated in future studies.

## Conclusions

Short-term clinical outcomes associated with resorbable P4HB mesh are comparable to permanent synthetic PP mesh in both umbilical and small to medium, routine ventral hernia repairs. Longer-term clinical follow-up is needed to expand on the clinical relevance of these short-term findings.

## Data Availability

The raw data supporting the conclusions of this article may be made available by the authors, without undue reservation.
